# A morphometric analysis of vegetation patterns in dryland ecosystems

**DOI:** 10.1098/rsos.160443

**Published:** 2017-02-15

**Authors:** Luke Mander, Stefan C. Dekker, Mao Li, Washington Mio, Surangi W. Punyasena, Timothy M. Lenton

**Affiliations:** 1 College of Life and Environmental Sciences, University of Exeter, Exeter EX4 4PS, UK; 2 Department of Environment, Earth and Ecosystems, The Open University, Milton Keynes MK7 6AA, UK; 3 Department of Environmental Sciences, Copernicus Institute of Sustainable Development, Utrecht University, PO Box 80115, Utrecht 3508 TC, The Netherlands; 4 Department of Mathematics, Florida State University, Tallahassee, FL 32306, USA; 5 Department of Plant Biology, University of Illinois, Urbana, IL 61801, USA

**Keywords:** vegetation patterns, morphology, morphometrics, ecohydrology, computational vision

## Abstract

Vegetation in dryland ecosystems often forms remarkable spatial patterns. These range from regular bands of vegetation alternating with bare ground, to vegetated spots and labyrinths, to regular gaps of bare ground within an otherwise continuous expanse of vegetation. It has been suggested that spotted vegetation patterns could indicate that collapse into a bare ground state is imminent, and the morphology of spatial vegetation patterns, therefore, represents a potentially valuable source of information on the proximity of regime shifts in dryland ecosystems. In this paper, we have developed quantitative methods to characterize the morphology of spatial patterns in dryland vegetation. Our approach is based on algorithmic techniques that have been used to classify pollen grains on the basis of textural patterning, and involves constructing feature vectors to quantify the shapes formed by vegetation patterns. We have analysed images of patterned vegetation produced by a computational model and a small set of satellite images from South Kordofan (South Sudan), which illustrates that our methods are applicable to both simulated and real-world data. Our approach provides a means of quantifying patterns that are frequently described using qualitative terminology, and could be used to classify vegetation patterns in large-scale satellite surveys of dryland ecosystems.

## 1. Introduction

**Figure 1.**
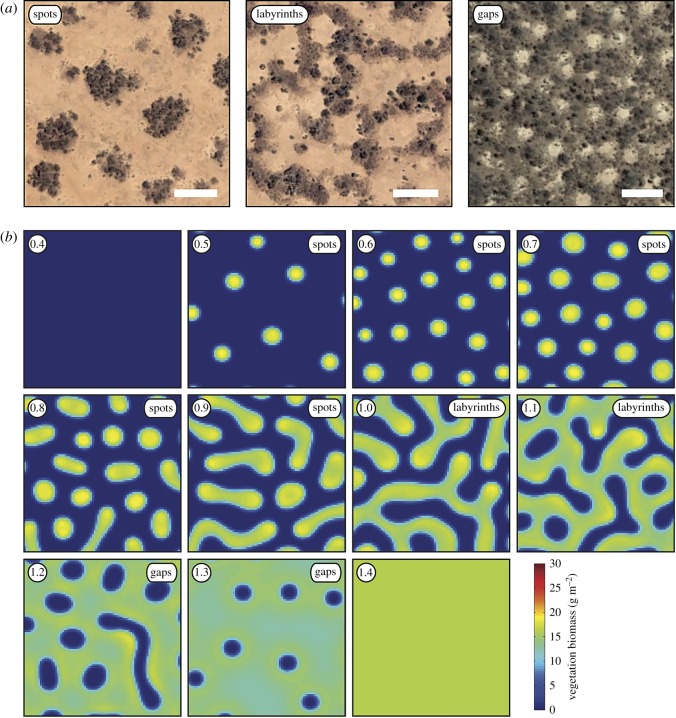
Examples of patterned vegetation recorded by satellite imagery (*a*) and produced by the computational model of [1] (*b*). At 0.4 mm of rainfall per day, the simulated land surface is bare ground devoid of vegetation, and at 1.4 mm of rainfall per day the land surface is covered with uninterrupted vegetation (*b*). Scale bars in (*a*) represent 50 m. Circular insets in the upper left-hand corner of each thumbnail in (*b*) show rainfall in mm per day.

**Figure 2.**
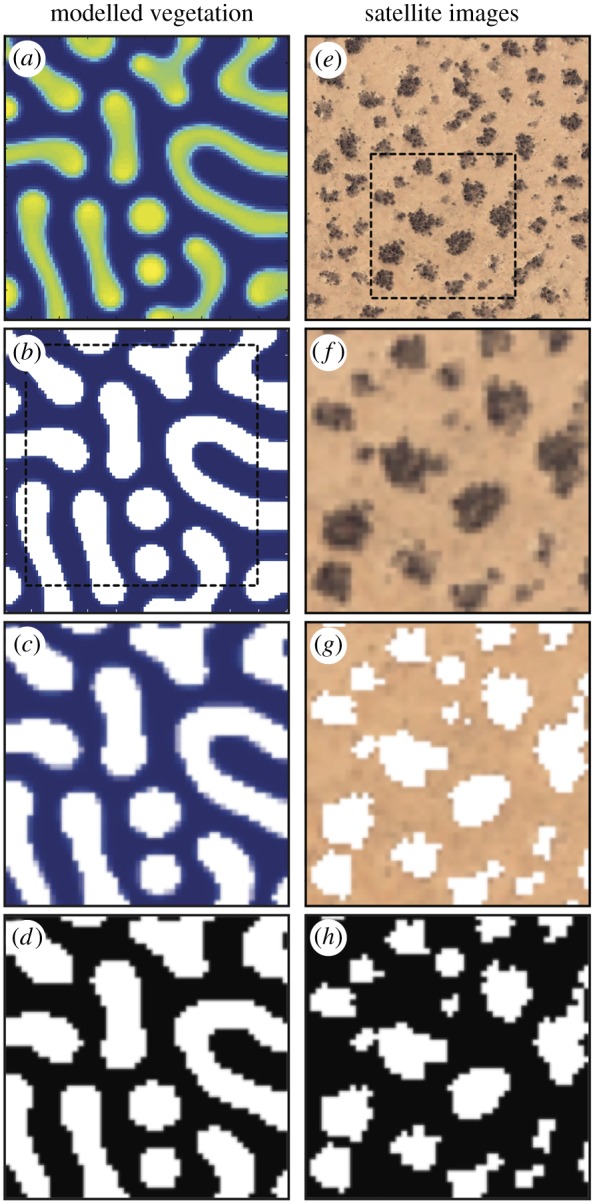
Thumbnails showing the image processing steps taken during the segmentation of modelled vegetation images (*a*–*d*) and satellite images (*e*–*h*). In the binary images (*d* and *h*), vegetation patches are represented by foreground (white) pixels, and bare ground interpatches are represented by background (black) pixels.

Vegetation in dryland ecosystems of Africa, North America, Australia and Asia often forms remarkable spatial patterns. These range from regular bands of vegetation alternating with bare ground, to vegetated spots and labyrinths, to regular gaps of bare ground within an otherwise continuous expanse of vegetation. These patterns can be observed in satellite imagery (figure 1*a*; [2–5]), and can be produced by activation–inhibition systems in computational models (see [6] for a review) (figure 1*b*; [2,7–11]). The development of spatial vegetation patterns in simulations follows a well-established sequence that is related to the amount of rainfall supplied to the land surface. At relatively high rainfall levels, the entire land surface is covered with vegetation, and as the rainfall progressively decreases vegetation patterns change from gaps (near continuous vegetation cover with small openings) to labyrinths (reticulate networks of vegetation) to spots (small patches of vegetation), and finally to bare ground (figure 1*b*; [10,12]). It is thought that these spatial vegetation patterns result from the enhanced infiltration of water into vegetated ground compared with bare ground, and/or extensive lateral root networks, both of which promote the growth of vegetation at very local scales but inhibit vegetation growth over a larger area because of competition for water [10,12]. Additionally, fast soil-water diffusion in porous sand results in an uptake–diffusion feedback, which requires only confined and not extensive lateral roots. This feedback has been used to simulate gap patterns in Namibia [13–15] and has been described more formally in [16].

Hysteresis is thought to be pervasive in these pattern-forming systems [17,18], and at very low rainfall levels spotted vegetation patterns and bare ground may represent alternative stable states [9,18,19]. Complete vegetation patch disappearance is accompanied by the loss of a functioning root network as well as the mechanism for enhanced soil-water infiltration, and re-colonization of the bare ground is only possible if the rainfall increases substantially above the level at which spotted vegetation patterns form [19]. As a result, it has been suggested that the formation of spotted vegetation patterns could indicate that collapse into a bare ground state is imminent (e.g. [18]), and the morphology of spatial vegetation patterns, therefore, represents a potentially valuable source of information on the proximity of regime shifts in dryland vegetation [9,12,18,20].

However, descriptions of spatial vegetation patterns are currently limited to qualitative descriptive nomenclature (e.g. figure 1*a*), Shannon entropy values [1] and Fourier analysis [2–5], and at present there is ‘no unique “measure” that captures all aspects of pattern morphology’ ([1, p. 9]). More holistic measures of vegetation patterning may help to describe regime shifts in simulated dryland vegetation with greater accuracy, and could also be used to classify vegetation patterns in large-scale field surveys of dryland ecosystems using satellite imagery (e.g. [4]).

To address this, we have developed quantitative morphometric methods to characterize spatial patterns in dryland vegetation. Our approach is based on algorithmic techniques that have been used to classify grass pollen grains on the basis of textural patterning [21], and involves using image processing to convert raw colour images of vegetation patterns to binary images, and constructing feature vectors to quantify the shapes in these binary images. These feature vectors can be thought of as numerical summarizations of the image properties, and are derived from measurements of subgraph centrality [22] and the Euler characteristic. Indices of centrality measure the relative importance of the vertices within a graph [22], and the Euler characteristic is a topological invariant: a property that is preserved throughout deformations of an object. We also investigate the nature of transitions between different vegetation patterns along a rainfall gradient by measuring the size of vegetation patches. We have analysed images of patterned vegetation produced by a computational model [1] (figure 1*b*) and a small set of satellite images from South Kordofan in South Sudan (following [4]) (table 1; figure 1*a*) in order to illustrate that our methods are capable of characterizing patterning in both simulated and real-world data.

**Table 1.** Summary of satellite images analysed in this study. Annual rainfall for each site from Bioclim (http://www.worldclim.org/bioclim).

pattern	imagery date	image number	elevation (m)	latitude	longitude	annual rainfall (mm)
gaps	11 Feb 2014	1	457	10°59′25.85″ N	28°15′28.19″ E	565
		2	457	11°00′02.58″ N	28°14′49.76″ E	562
		3	447	10°56′47.86″ N	28°17′01.65″ E	572
		4	446	10°55′11.84″ N	28°16′52.41″ E	576
		5	446	10°54′56.22″ N	28°17′22.44″ E	579
		6	446	10°52′57.58″ N	28°19′27.70″ E	585
		7	447	10°51′35.48″ N	28°20′19.18″ E	591
		8	444	10°50′22.15″ N	28°21′40.36″ E	596
		9	438	10°46′31.83″ N	28°15′19.32″ E	594
		10	440	10°49′41.37″ N	28°16′38.64″ E	588
labyrinths	20 Mar 2004	1	466	11°17′16.24″ N	27°59′29.92″ E	499
		2	464	11°16′53.98″ N	27°59′27.60″ E	500
		3	461	11°16′00.09″ N	27°59′07.92″ E	501
		4	461	11°15′58.79″ N	27°58′45.08″ E	502
		5	463	11°16′23.97″ N	27°58′48.12″ E	501
		6	461	11°16′10.46″ N	27°58′25.02″ E	501
		7	462	11°16′33.08″ N	27°58′27.78″ E	499
		8	462	11°16′51.13″ N	27°58′25.37″ E	499
		9	464	11°18′07.09″ N	27°58′01.50″ E	494
		10	465	11°18′53.85″ N	27°58′32.25″ E	494
spots	20 Mar 2004	1	476	11°34′27.91″ N	27°56′19.72″ E	456
		2	476	11°34′50.81″ N	27°56′17.93″ E	455
		3	476	11°34′47.73″ N	27°56′41.44″ E	455
		4	477	11°34′23.54″ N	27°56′43.19″ E	457
		5	478	11°34′20.83″ N	27°57′07.97″ E	458
		6	477	11°35′00.28″ N	27°57′10.23″ E	454
		7	477	11°34′54.53″ N	27°56′37.02″ E	455
		8	478	11°35′36.83″ N	27°55′30.32″ E	453
		9	477	11°34′47.42″ N	27°56′46.00″ E	455
		10	473	11°31′09.87″ N	27°56′06.81″ E	464

## 2. Material and methods

### 2.1. Image library

This study is based on images of patterned vegetation that have been produced by a computational model and that have been extracted from satellite imagery. The dataset of modelled vegetation images consists of 45 images that were produced by running the computational model of Konings *et al*. [1] (without rainfall-related feedback and over a uniformly flat topography) five times with different initial vegetation at nine rainfall intervals: 0.5 mm per day, 0.6 mm per day, 0.7 mm per day, … 1.3 mm per day (figure 1*b*). In these simulations, the grid size was 100 × 100 cells with each cell measuring 2 × 2 m, and vegetation biomass was recorded in grams per square metre. Each simulation was run over 100 000 days in order to reach a stable solution. The dataset of satellite images consists of 10 images of spotted patterns, 10 images of labyrinth patterns and 10 images of gap patterns (pattern nomenclature follows [1]; figure 1*a*; table 1). These images were collected using Google Earth, and are from the West of South Kordofan in South Sudan (following [4]). Each image was collected at an eye altitude of 1 km. The climate in this region is semi-arid, with annual rainfall typically ranging from 370 to 600 mm [4]. The growth of vegetation in this region occurs during the short rainy season from June to September, and it is during this period that 89% of the annual rainfall occurs (see [4]). The satellite images of spotted and labyrinth patterns are dated 20 March 2004, and the images of gap patterns are dated 11 February 2014. All satellite images record vegetation during the dry season.

### 2.2. Image segmentation

For modelled vegetation, each raw image of model output (figure 2*a*) was thresholded to 5 g of biomass per square metre (figure 2*b*). Each thresholded image was then scaled by a factor of 0.17 using bilinear interpolation in order to reduce the number of pixels in each image, and a 50 × 50 pixel window was manually cropped from each image (figure 2*c*). This 50 × 50 pixel window was converted into a binary image by thresholding (figure 2*d*). Each 50 × 50 window measures 161 × 161 m. For real-world vegetation, a single 350 × 350 pixel window was manually cropped from each raw satellite image (figure 2*e*). The resolution of each of these windows was reduced to 50 × 50 pixels, measuring 283 × 283 m (figure 2*f* ) and then thresholded using pixel intensity values to delineate vegetated patches (figure 2*g*). This 50 × 50 pixel image was converted into a binary image by thresholding (figure 2*h*). These 50 × 50 images measure 283 × 283 m. In both simulated and real-world images, vegetation patches are represented by foreground (white) pixels, and bare ground interpatches are represented by background (black) pixels (figure 2*d*,*h*).

### 2.3. Quantitative characterization of vegetation patterns

We quantitatively characterized simulated and real-world vegetation patterns by deriving a 20-dimensional feature vector from each binary image in our dataset. This feature vector describes the shape and patterning of the vegetation. We began by forming a graph from each 50 × 50 pixel binary image, with the pixels in each image as the vertices in each graph. Two vertices were connected with an edge if they fell within the 3 × 3 neighbourhood of each other. We experimented with graphs formed from foreground pixels that represent vegetation patches (white pixels in figure 2*d*,*h*), and with graphs formed from background pixels that represent bare ground interpatches (black pixels in figure 2*d*,*h*).

These experiments indicated that for simulated vegetation, graphs formed from foreground (vegetated) pixels produced a clearer characterization of the patterning than graphs formed from background (bare ground) pixels. The opposite was true for vegetation patterning recorded by satellite imagery, and graphs formed from background pixels produced clearer characterization of these real-world patterns. Accordingly, we have characterized simulated vegetation patterns on the basis of foreground shapes and characterized real-world vegetation patterns on the basis of background shapes.

Following Mander *et al*. [21], we then ranked the vertices in each graph (the pixels in each image) using subgraph centrality (SC) [22], which can be defined as follows. For a vertex *v* and a non-negative integer *ℓ*, let *µ_ℓ_*(*v*) denote the number of closed walks of length *ℓ* starting at *v*. Then, the centrality of *v* is defined as

2.1 $$\text{SC}(\nu )=\sum_{\ell =0}^{\infty }\frac{\mu_{\ell }(\nu )}{\ell !},$$

a weighted sum that can be computed in terms of the eigenvalues and eigenvectors of the adjacency matrix of the graph [22].

**Figure 3.**
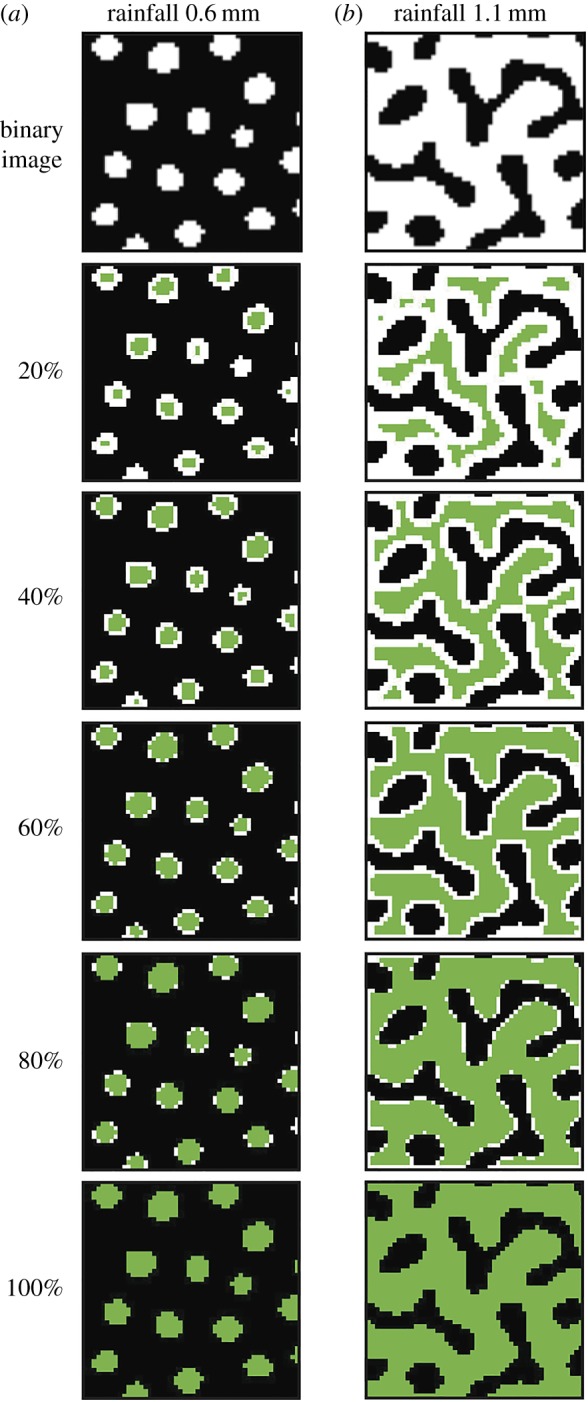
Thumbnails showing sequences of expanding subregions of graphs derived from images of modelled vegetation at 0.6 mm rainfall per day (*a*) and 1.1 mm rainfall per day (*b*). In these examples, the graphs were derived from the foreground (white) pixels of each binary image, and the vertices are shown as green pixels. The expanding subregions in these examples begin with the vertices that were ranked in the top 20% according to SC, and lower-ranked vertices are added in 20% increments until 100% of the vertices are present.

We then formed a sequence of expanding subregions of each graph, beginning with the vertices that were ranked in the top 5% according to SC, and adding lower-ranked vertices in 5% increments (figure 3). These subregions are composed of a number of connected components. In figure 3*b*, for example, the subregion with 20% of the vertices displayed contains 11 connected components, and the subregion with 80% of the vertices displayed contains a single connected component. To describe the structure of each subregion, we formed a graph (*G*) from each connected component using a neighbouring relation to connect vertices with an edge. We then subtracted the number of edges (*E*) from the number of vertices (*V*) in each graph in order to calculate the Euler characteristic (*χ*) of each subregion. We define the Euler characteristic as:

2.2 χ(G)=V−E,

a definition that lacks faces. The values of *χ* for each sequence of expanding subregions were recorded in a 20-dimensional feature vector.

## 3. Results

### 3.1. Vegetation pattern morphology

**Figure 4.**
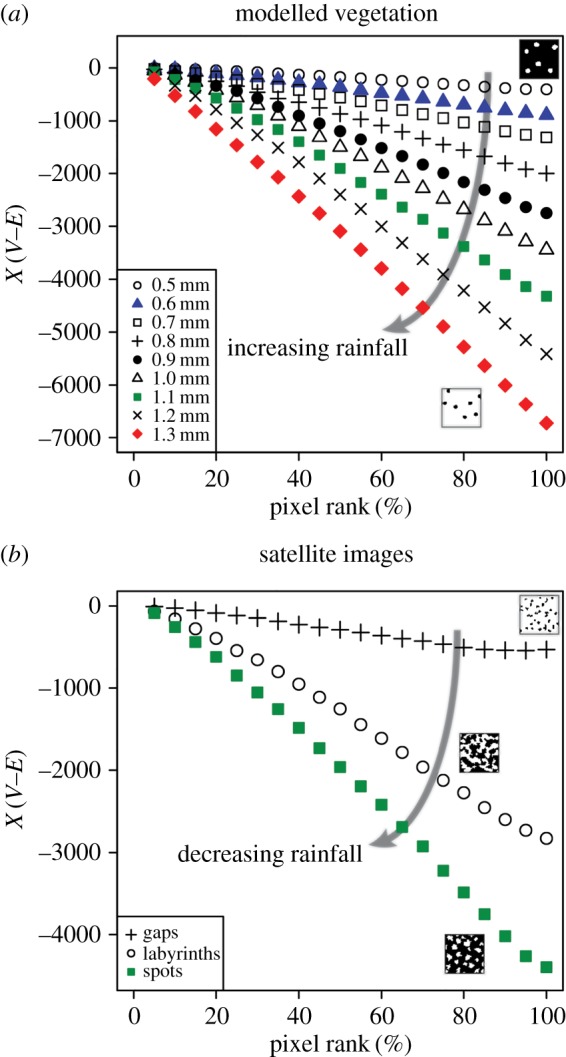
Scatterplots showing the 20-dimensional feature vectors derived from each image of modelled (*a*) and real-world vegetation (*b*) that is shown in figure 1. Each sequence of 20 Euler characteristic (*χ*-values, which were calculated by subtracting the number of edges (*E*) from the number of vertices (*V*) in each subregion, represents a single feature vector. Binary image thumbnails in (*a*) show an example of a spotted pattern formed at 0.5 mm rainfall per day (top right) and a gap pattern formed at 1.3 mm rainfall per day (lower right). Thumbnails in (*b*) show examples of gap, labyrinth and spotted patterns. Vegetation patches in these thumbnails are represented by white pixels; bare ground interpatches are represented by black pixels.

These feature vectors are characterized by values of *χ* that decrease with increasing pixel rank (figure 4). Feature vectors that describe simulated vegetation patterns tend to increase in slope as the rainfall increases (figure 4*a*). Vegetation patterns composed of sparsely distributed circular to sub-circular patches of vegetation are typically characterized by a relatively shallow feature vector, whereas vegetation patterns composed of a single continuous shape are typically characterized by a relatively steep feature vector (figure 4*a*).

Feature vectors that describe real-world vegetation patterns recorded in satellite imagery show the opposite trend, and tend to increase in slope as the rainfall decreases (figure 4*b*). This is because the feature vectors that describe simulated vegetation were derived from white foreground pixels, whereas the feature vectors that describe real-world vegetation were derived from black background pixels. Gap patterns, which formed at sites receiving relatively high rainfall in our dataset (table 1), are typically characterized by a relatively shallow feature vector, but spotted patterns, which formed at sites receiving relatively low rainfall in our dataset (table 1), are typically characterized by a relatively steep feature vector (figure 4*b*).

**Figure 5.**
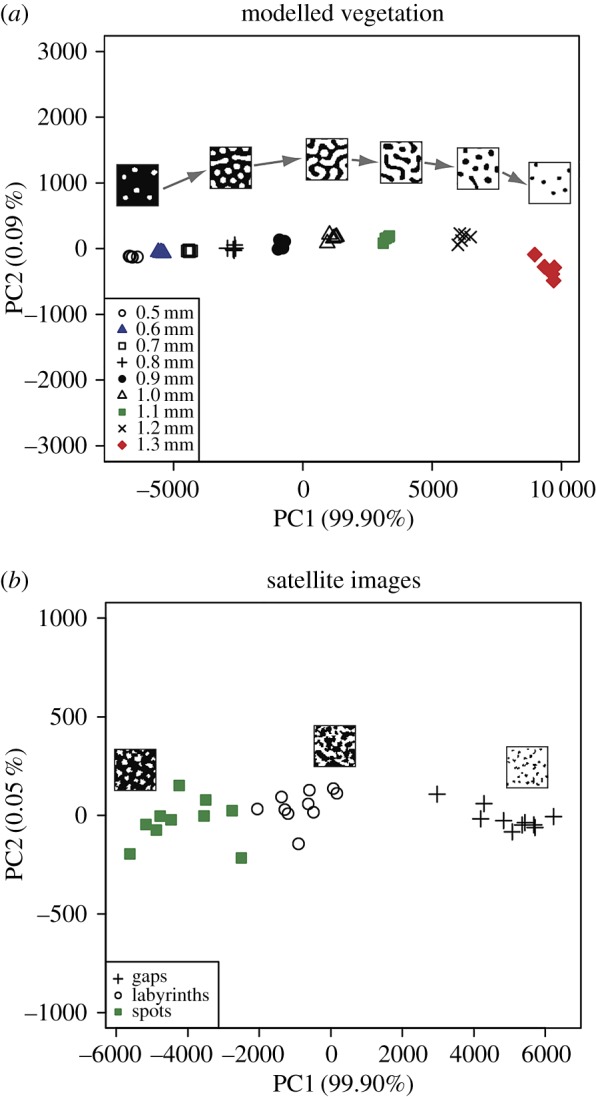
Scatterplots showing the results of principal component analyses of the feature vectors used to describe modelled vegetation patterns (*a*) and vegetation patterns observed in satellite imagery (*b*). Vegetation patches in binary image thumbnails are represented by white pixels; bare ground interpatches are represented by black pixels. The sequence of thumbnails from left to right in (*a*) is as follows: 0.5 mm rainfall per day, 0.8 mm rainfall per day, 1.0 mm rainfall per day, 1.1 mm rainfall per day, 1.2 mm rainfall per day and 1.3 mm rainfall per day. The first principal component explains almost all the variance (greater than 99%) in the analyses of modelled (*a*) and real-world (*b*) vegetation patterns.

We used principal component analysis (PCA) to reduce the dimensions of each feature vector and to graphically compare the images of patterned vegetation in our dataset. The first principal component explains the vast majority of the variance (up to 99.9%) in both the modelled and real-world vegetation data (figure 5), and therefore the other principal components were not analysed further. For modelled vegetation, each of the nine rainfall intervals is clearly distinguished and there is no overlap between images from different rainfall intervals (figure 5*a*). The images of patterned vegetation produced at 0.5–0.7 mm rainfall per day plot closely to one another and are characterized by low PC1 values (figure 5*a*). The images from the rainfall interval 1.2–1.3 mm per day are characterized by high PC1 values, and are separated clearly both from each other and from images produced at lower rainfall intervals (figure 5*a*). All of the three pattern types observed in satellite imagery are distinct from one another, and there is no overlap of data points from different patterns (figure 5*b*). Satellite images of spotted patterns and labyrinth patterns lie closely together in the PCA scatterplot, and gap patterns plot in a distinct cluster characterized by high PC1 values (figure 5*b*).

### 3.2. Vegetation patch size

**Figure 6.**
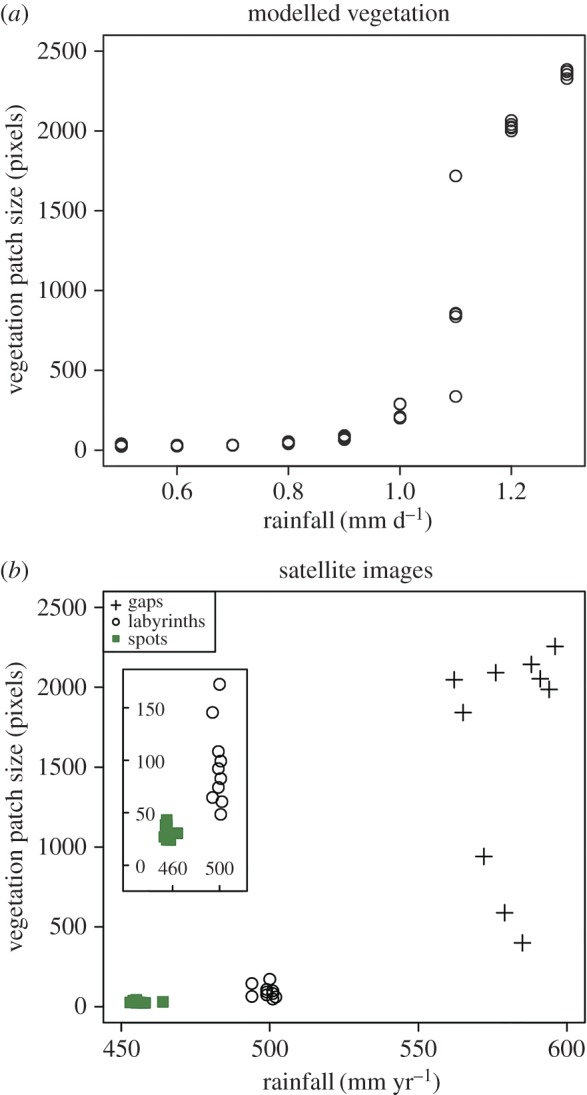
Scatterplots showing the relationship between vegetation patch size and rainfall in modelled vegetation patterns (*a*), and the variations in vegetation patch size among three vegetation pattern types observed in satellite images (*b*). Inset in (*b*) shows the size of vegetation patches in labyrinth and spotted vegetation in more detail. Vegetation patch size calculated from binary images (see Material and methods) and reported in pixels. Where an image contained more than one vegetation patch (e.g. figure 1*b* 0.6 mm rainfall per day), size is reported as the average of all the vegetation patches present in the image.

The size of the modelled vegetation patches varies at different levels of rainfall. At low rainfall levels (0.5–0.7 mm per day), vegetation patch size ranges from 24 to 40 pixels, and as the rainfall increases to 1.0 mm per day, vegetation patch size increases to between 200 and 289 pixels (figure 6*a*). Vegetation patch size at 1.1 mm of rainfall per day is extremely variable and ranges from 336 to 1718 pixels (figure 6*a*). The size of the modelled vegetation patches at 1.2–1.3 mm of rainfall per day ranges from 2000 to 2385 pixels (figure 6*a*). The abrupt increase in vegetation patch size in the interval spanning 1.0–1.2 mm per day of rainfall reflects a threshold transition from vegetation patterns composed of a number of individual patches, to vegetation patterns composed of a single continuous labyrinth of vegetation (figure 1*b*).

There are also differences in the size of real-world vegetation patches recorded by satellite imagery. The spotted patterns, which formed at localities receiving 453–464 mm of annual rainfall (table 1), are composed of patches measuring between 24 and 43 pixels in size (figure 6*b*). Labyrinth patterns, which formed at localities receiving 494–502 mm of annual rainfall (table 1), are composed of patches that measure 48–172 pixels (figure 6*b*). The gap patterns formed at localities receiving annual rainfall between 562 and 596 mm (table 1) and the size of the vegetation patches at these localities is quite variable, ranging from 400 to 2256 pixels (figure 6*b*).

## 4. Discussion

Shapes are distinguished by their complements, and in the context of the binary images in our dataset (e.g. figures 3 and 5), the foreground shapes formed of white pixels are reflected in the background shapes formed of black pixels and vice versa. We have made use of this observation in our analyses by characterizing modelled vegetation on the basis of foreground shapes, and characterizing real-world vegetation on the basis of background shapes. This was done primarily in order to achieve the clearest possible separation between pattern types in PCA plots. We suspect that the clear characterization of modelled vegetation patterns using foreground (vegetated) pixels might be related to the fact that these simulated vegetation patches are displayed as uniform green–yellow pixels with distinct and regular borders (figure 1*b*). By contrast, the vegetation patches in real-world images are frequently quite small, particularly for spotted patterns (figure 2*e*,*f*), and have margins that are somewhat irregular (e.g. figure 1*a*).

However, it also reflects a degree of flexibility and economy in our approach. For example, in related work on the classification of grass pollen, the surface patterning of certain species was described by feature vectors derived from weighted graphs in which edges connecting two foreground or two background pixels were weighted differently to foreground–background transition edges [21], but this degree of complexity was not required for the shapes we have investigated here. In this context, our results indicate that our approach to the characterization of shapes (figure 3) is applicable across scales, from the nanoscale features of pollen morphology [21] to the landscape-scale aspects of vegetation patterns (figure 1*a*).

Vegetation patch size is thought to be a key aspect of vegetation pattern morphology in dryland ecosystems, and patch size distributions have been proposed as a warning signal for the onset of desertification, for example [23,24]. The importance of vegetation patch size is emphasized by the abrupt increase in simulated vegetation patch size at 1.1 mm of rainfall per day (figure 6*a*). This is the only simulated evidence of a regime shift (*sensu* [19]) in vegetation pattern morphology in our dataset, and it reflects a transition from vegetation patterns composed of several relatively small individual patches, to vegetation patterns composed of a single relatively large continuous labyrinth of vegetation at 1.0–1.2 mm of rainfall per day (figures 1*b*, 6*a*). This transition resembles a percolation transition, leading to the formation of a spanning cluster in a network (e.g. [12,25]), and previous studies of simulated dryland vegetation patterns in terms of percolation theory have noted similar threshold behaviour in vegetation patch size [26]. There is considerable overlap in vegetation patch size at low rainfall levels (0.5–0.7 mm per day) (figure 6*a*; see also figure 1*b*), and this highlights that vegetation patch size alone does not distinguish all of the nine rainfall intervals studied here.

Our approach to the characterization of spatial patterns in dryland vegetation represents an additional measure of vegetation pattern morphology, which complements existing ways of describing of these patterns using descriptive nomenclature (e.g. figure 1*a*), Fourier analysis (e.g. [2–5]) and Shannon entropy [1]. Fourier analysis and spatial correlation analysis are particularly well suited to the analysis of these self-organized patterns because they are characterized by the spatially explicit distribution of structural nodes that lead to a specific vegetation pattern wavelength, which, in turn, gives rise to a distinctive periodicity (e.g. [2]). Some recent analyses of vegetation patterns have been undertaken in a spatially explicit framework (e.g. [13,14]). However, our analysis, which is focused on the quantitative description of pattern morphology, lacks such a dynamical and explicitly spatial component in the sense that it does not explicitly describe how pattern morphology changes dynamically from one point in space to another. Additionally, our dataset of simulated vegetation patterns consists of images produced at discrete rainfall intervals, and our dataset of satellite images consists of individually separate images of vegetation patterns. The discrete nature of this dataset limits our ability to examine dynamic spatially explicit changes in vegetation pattern morphology.

Nevertheless, since subgraph centrality provides a measure of both the local and the global connectivity of a graph [22], it can capture some spatial information from continuous shapes such as the bare ground between patches of spotted vegetation (black pixels in figure 2*h*). An example of such information might be the spatial distribution of network motifs, which can be characterized using subgraph centrality [22], and analyses of motif distribution in spatially embedded networks (e.g. [27]) could be undertaken in future work. This could also include a quantitative comparison of how changes in vegetation pattern morphology across water stress gradients are captured by existing Fourier techniques (e.g. [2–5]) and by the methods we have developed in this paper.

The quantitative description of shapes and patterns using computational image analysis is increasing the accuracy and speed with which biological objects are analysed and classified in disciplines such as taxonomy and molecular biology (e.g. [28–30]), and our results indicate that a similar approach can be usefully applied to images generated as part of research programmes in Earth science and ecology. In the context of spatial vegetation patterns in dryland ecosystems, the approach we have developed in this paper adds to the growing toolbox of methods for analysing these patterns (e.g. [31]), and could be implemented within models such as those of [1] and [32] to assess pattern morphology dynamically rather than at discrete rainfall intervals (cf. figure 1*b*). In this context, future work could explore the degree to which our characterization of vegetation pattern morphology could be used as an early warning signal of vegetation change. Additionally, our methods could be used to classify vegetation patterns in large-scale satellite surveys of dryland ecosystems (e.g. [2–4,31]).

## Data accessibility

The images on which this article is based have been uploaded to the Dryad Digital Repository: http://dx.doi.org/10.5061/dryad.5d19r [33].
